# Assessment of tilt and decentration of a 4-point scleral fixated intraocular lens using an innovative digital measurement methodology

**DOI:** 10.1186/s40942-025-00690-5

**Published:** 2025-06-20

**Authors:** Denise Pardini, Monica MS Matsumoto, Marina Roizenblatt, Ana Marisa Branco, Paulo Schor, Norma Allemann, Michel Farah, André Maia, Maurício Maia

**Affiliations:** 1 Department of Ophthalmology, Federal University of São Paulo, Denise Pardini 822, Botucatu St. Vila Clementino, São Paulo, SP Brazil; 2 Electronics Engineering Division, Aeronautics Institute of Technology, São José dos Campos, Brazil

**Keywords:** Tilt, Decentration, 4-support intraocular lens, Scleral fixation

## Abstract

**Background:**

The study underscores the importance of conducting objective measurements of intraocular lens (IOL) tilt and decentration over time to provide evidence of IOL stability. The goals were to validate a new method for measuring tilt and decentration of secondary implants and evaluate the postoperative functional and anatomic outcomes of 4-support IOL scleral fixation.

**Methods:**

This prospective study (7 patients, seven eyes) was conducted from October 2020 to April 2021 at the Department of Ophthalmology, Federal University of São Paulo, São Paulo, and Technological Institute of Aeronautics, São José dos Campos, Brazil. The patients underwent combined vitrectomy and implantation of a 4-point scleral fixation IOL. The subjective refraction with the logarithm of the minimum angle of resolution (logMAR) best-corrected visual acuity (BCVA), slit-lamp examination, Goldmann applanation tonometry, wide-field retinography, and Scheimpflug corneal tomography and topography were performed preoperatively and postoperatively. Macular optical coherence tomography (OCT) was performed to assess the presence of macular edema. Anterior-segment OCT and ultrasound biomicroscopy (UBM) were performed to measure IOL positioning. The method for measuring IOL positioning was validated. Surgery outcomes included BCVA, refraction, and IOL tilt and decentration.

**Results:**

High intra-class correlations (0.781 to 0.999) were found for the intra- and inter-observer measurements of the UBM and OCT images. Measurements obtained using the scleral spur (SS) as an anatomic reference are comparable to those obtained using the iris pigmented epithelium (IPE) plane as a reference. Conversely, the measurements taken with OCT images cannot be compared to those obtained with UBM images. The logMAR BCVA improved from 0.59 ± 0.13 preoperatively to 0.24 ± 0.34 at 6 months postoperatively (*P* = 0.001). The mean tilt using anterior-segment OCT was 1.15 ± 1.26 degrees. The mean decentration on UBM images was 0.58 ± 0.63 mm; no significant changes in IOL position were observed between 1 and 6 months postoperatively.

**Conclusion:**

The methodology exhibits strong reproducibility and enabled valid comparisons of tilt and decentration across the SS and IPE. Four-support IOL scleral fixation yields favorable surgical outcomes with physiologic IOL position and maintains stability throughout the follow-up period.

**Trial registration:**

The Federal University of São Paulo institution’s Research Ethics Committee reviewed and approved this study protocol (Approval Number, 4.261.258).

**Supplementary Information:**

The online version contains supplementary material available at 10.1186/s40942-025-00690-5.

## Introduction

The surgical approach for patients with aphakia and insufficient capsular support continues to be debated. Presently, numerous surgical techniques are available for secondary intraocular lens (IOL) implantation in the anterior and posterior ocular chambers. Nevertheless, no single technique has a definitive advantage over the others [1].

In the past decade, the 4-point IOL scleral fixation technique has gained popularity among surgeons for addressing aphakia because of its favorable visual and anatomic outcomes [2–4]. This technique is believed to provide enhanced stability over time due to the four fixation points and the use of Gore-Tex sutures at the sclera; however, the lack of published studies cannot substantiate this claim.

Efforts to accurately measure secondary implantation IOL positioning in the eye have been performed with initial attempts using ultrasonic biomicroscopy (UBM) techniques in the early 1990s [5]. UBM can provide images of the anterior and posterior ocular chambers, which facilitates improved visualization and measurement of IOL positioning postoperatively. However, its limitations include limited examination availability, high cost, and reliance on well-trained medical examiners [6].

The advent of anterior-segment optical coherence tomography (AS-OCT) has enabled the measurement of IOL positioning with comparable accuracy [7]. As its popularity rises, it has become more accessible to physicians assessing IOL placement, and high-resolution images can be captured easily by non-medical technicians. Nonetheless, UBM and AS-OCT measurements depend on the instrument’s calipers, and if images are obtained from another service, it may not be possible to perform measurements and obtain important information.

The objectives of this study were to propose a standardized method for evaluating IOL positioning over time with a machine-free method to measure the tilt and decentration, using an algorithm applied to an image processor and to measure the anatomic and functional surgical outcomes of the scleral fixation of a 4-point IOL.

## Methods

The Ethics Committee of the Federal University of São Paulo approved this prospective study that was conducted according to the principles of the Declaration of Helsinki. All patients provided informed consent. The study was conducted from October 2020 to April 2021. Eight eyes of eight aphakic patients underwent combined vitrectomy and implantation of a 4-point scleral fixation IOL using Gore-Tex sutures. One patient passed away during follow-up due to complications related to COVID-19, resulting in a final cohort of seven patients.

The patients were followed on postoperative days 1, 7, 30, 90, and 180. The inclusion criteria were aphakia and absence of capsular support; the exclusion criteria were the presence of any macular or corneal disease, retinal detachment, advanced glaucoma and/or history of refractive surgery. All patients were aphakic due to complications from anterior segment surgery. The preoperative data collection included demographic data, previous surgeries, and the past ocular history.

Solix AS-OCT (Optovue, Fremont, CA, USA) was conducted 1, 3, and 6 months postoperatively through a dilated pupil to analyze the IOL position. UBM (VuMAX, Sonomed Escalon, Lake Success, NY, USA) also was performed at months 1 and 6 to compare the positioning analysis with that obtained from AS-OCT, considering that UBM is the gold standard for retro-pupillary IOL positioning analysis. Images of the ocular anterior segment were obtained at the horizontal (0° to 180°) and vertical (90° to 270°) meridians.

IOL power calculations were determined using the Sanders Retzlaff Kraff theoretical formula, with non-contact optical biometry measurements with IOLMaster 500 (Carl Zeiss Meditec AG, Jena, Germany) and immersion ultrasound biometry (Aviso, Quantel Medical, Clermont-Ferrand, France), targeting a refraction of about − 0.50 diopter (D), with scleral fixation 3.5 mm from the corneal limbus. Scheimpflug corneal tomography and topography (Pentacam AXL, Oculus Optikgerate GmbH, Wetzlar, Germany) was also performed at 30, 90 and 180 days postoperatively to evaluate corneal astigmatism and pachymetry. The prediction error was calculated as the difference between the 6-month postoperative refraction expressed as the spherical equivalent and the predicted target refraction. Surgically induced astigmatism (SIA) was calculated using the online calculator (https://sia-calculator.com) [8].

**Fig. 1:**
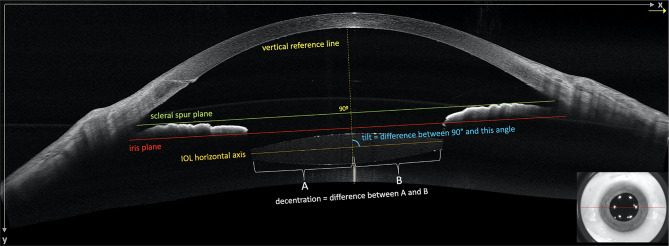
Horizontal scan of anterior segment optical coherence tomography (OCT) highlighting the reference lines used in the study. The scleral spur (SS) plane (green): line connecting the SSs; iris plane (red): line connecting the iris pigmented epithelium; vertical reference line (dotted yellow): line drawn perpendicularly through the midpoint of the horizontal reference; intraocular lens (IOL) horizontal axis (orange): line dividing the IOL into equal halves and A and B (white) sections of the optical zone of the lens positioned to the left and right of the visual reference line, respectively

To validate the new measurement method, two experienced retina specialists performed all tilt and decentration assessments. The first examiner performed the initial measurements and repeated them after 7 days for the intra-examiner comparison; the second observer conducted all measurements once for the inter-examiner comparison. Tilt measurements were derived from the AS-OCT and UBM images at 6 months postoperatively, using the scleral spurs (SS) and the plane of the iris pigment epithelium (IPE) as anatomic landmarks, as established in the literature (Fig. 1) [7, 9–11]. Decentration assessments were conducted exclusively using UBM images due to the limited visibility of the entire optical zone of the IOL in the AS-OCT images and using the same anatomic references. IOL stability was evaluated through tilt measurements from the AS-OCT images at months 1, 3, and 6 postoperatively alongside decentration evaluations from UBM images at months 1 and 6 postoperatively.

### Surgical technique

The same surgeon (DPM) performed all surgeries. Khan and colleagues described the surgical technique for implanting the Akreos AO60 IOL (Bausch & Lomb, Bridgewater, NJ, USA) fixated with Gore-Tex CV-8 sutures in 2015 [2]. In 2022, our team made modifications that were applied in this study [3]. None of the patients required additional procedures postoperatively, such as internal limiting membrane peeling, laser treatments, perfluorocarbon injection, or tamponade.

### Algorithm Pardini-Matsumoto

**Fig. 2:**
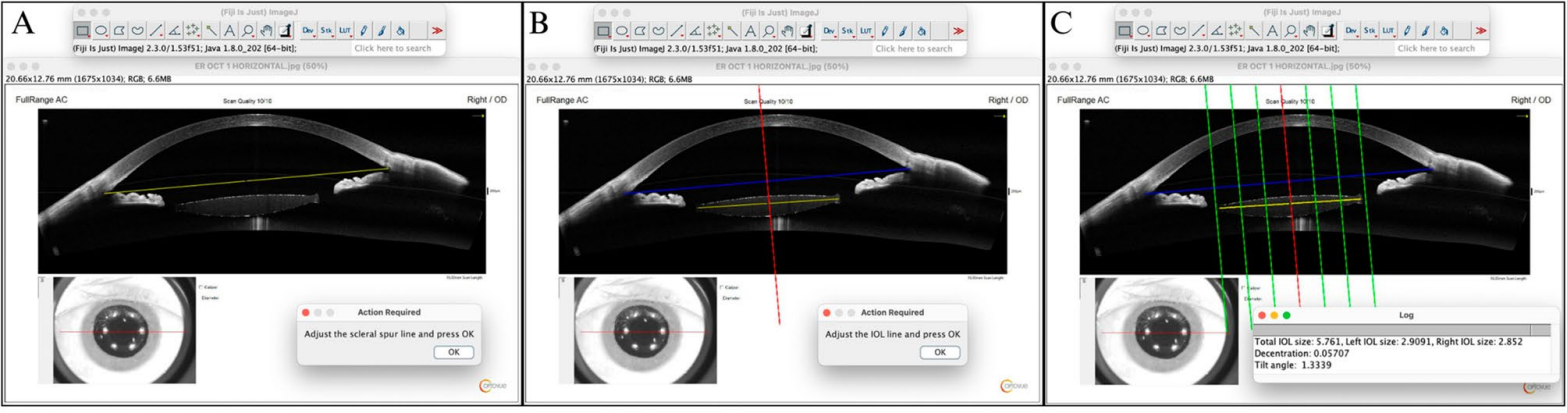
A step-by-step explanation for using the algorithm. (**A**) When the algorithm is initiated, a dialogue box prompts the examiner to position the first reference line in the eye, with two options: placing the line at the scleral spur or the plane of the iris pigment epithelium. After this placement, the examiner clicks OK, which automatically generates a perpendicular line at the midpoint of the first reference line. (**B**) A second dialogue box then appears asking the examiner to position the second reference line at the intraocular lens (IOL) horizontal axis and press OK again. (**C**) Following confirmation, the algorithm automatically generates values for tilt (degrees) and decentration (mm) and three lines are displayed to the left and right of the vertical reference line (red), spaced at 1, 2, and 3 mm

Our team developed two algorithms within the Fiji-ImageJ^®^ image processor to measure the IOL position (Video 1, Fig. 2). Fiji-Image J^®^ is a Java-based image processing program created by the National Institutes of Health (NIH), Bethesda, MD, USA, and the Laboratory for Optical and Computational Instrumentation (LOCI, University of Wisconsin, Madison, WI, USA). The first algorithm was designed for measurements in the UBM VuMAX^®^ images, while the second was tailored for AS-OCT images from the Solix^®^ equipment. Each algorithm accounts for distinct characteristics, considering the different levels of zoom onto the ocular structures to ensure accurate measurements.

To use the algorithm, users must download the Fiji-Image J^®^ software from the NIH website at https://imagej.nih.gov/ij/download.html. After downloading and opening the software, the algorithm file and the image file to be processed should be dragged into ImageJ, followed by running the algorithm.

To calculate the tilt, the algorithm subtracts the 90° angle between the horizontal reference (SS or IPE) and the vertical reference line (VRL) from the angle between the VRL and the IOL horizontal axis (Fig. 1). For decentration calculations, the measurement of the IOL horizontal axis was subtracted from each side of the VRL (Fig. 1). Three lines also are displayed to the left and right of the VRL, spaced at 1, 2, and 3 mm (Fig. 2C). This feature enables analysis when the optical zone of the IOL is not fully visible in the AS-OCT image, with the distance between the first and last line spanning 6 mm, matching the optical zone size of the IOL. When the IOL edges are between lines 2 and 3, this indicates the IOL is centered or up to 1 mm off-center. If one edge is between lines 2 and 1, the IOL may be up to 2 mm off-center, and if an edge is between line 1 and the VRL, the IOL may be up to 3 mm off-center.

### Statistical analysis

The sample power was calculated using PASS 14 (Power Analysis and Sample Size System) (NCSS, LLC, Kaysville, UT, USA) to assess the significance of the intra-class correlation via the F-test. A significance level of 5% was used for all statistical tests. The reproducibility of tilt and decentration between and within observers was assessed using the intra-class correlation, in which values close to 1 indicate high reproducibility.

The data were analyzed using means and standard deviations, and the intervention’s effect on ocular characteristics was assessed with repeated measures analysis of variance. The data normality was assessed using the Kolmogorov-Smirnov test. When mean differences were found, distinct groups were identified through multiple comparisons with Bonferroni correction. Analyses were conducted using SPSS 20.0 (SPSS Inc., Chicago, IL, USA) STATA 17 (Stata Corporation, College Station, TX, USA) statistical software.

## Results

### Methodology validation

Table 1 shows the high intra-class correlation analysis of the final tilt measurements for the intra-observer and inter-observer assessments, across the iris and SS, horizontally and vertically, and by UBM and AS-OCT, with correlation coefficients ranging from 0.781 to 0.996 (*P* < 0.005). An exception was the vertical scan position in the AS-OCT images in the SS, which showed a moderate intra-class correlation for the intra-observer measurements (*P* = 0.007). In contrast, the decentration measurements obtained from the UBM images displayed high intra-class correlation across all conditions, with coefficients ranging from 0.821 to 0.995 (*P* < 0.005).

Table 2 shows the intra-class correlation of the final tilt and decentration measurements by comparing the results obtained using the SS as a reference against those using the IPE as a reference in the vertical and horizontal scanning positions. The analysis showed a high intra-class correlation ranging from 0.838 to 0.995 (*P* < 0.005) across all time points and positions, except for the inter-observer measurements post-UBM in the horizontal position, which exhibited no significant correlation (*P* = 0.065). Additionally, when comparing the final tilt measurements between OCT and UBM, no intra-class correlation was observed.

A sample of 7 patients allows for the evaluation of the significance of the intraclass correlation using the F-test, with a significance level of 5% and a power of 80.7% for an estimated intraclass correlation of 0.78. Increasing the value of the intraclass correlation enhances the sample power.

**Table 1:** Intra-class correlation of the final Tilt and decentration measurements by image type, ocular anatomic reference, and scan position

Tilt measurements							
Image type/anatomic reference	Horizontal				Vertical		
	Intra-class correlation	95% Confidence interval	*P* value		Intra-class correlation	95% Confidence interval	*P* value
AS-OCT							
Scleral spur							
Intra-observer	0.973	0.868–0.995	< 0.001		0.781	0.227–0.958	0.007
Inter-observer	0.974	0.874–0.995	< 0.001		0.885	0.524–0.979	0.001
Iris							
Intra-observer	0.992	0.958–0.999	< 0.001		0.965	0.833–0.994	< 0.001
Inter-observer	0.934	0.701–0.988	< 0.001		0.970	0.857–0.995	< 0.001
UBM							
Scleral spur							
Intra-observer	0.824	0.338–0.967	0.003		0.994	0.970–0.999	< 0.001
Inter-observer	0.950	0.769–0.991	< 0.001		0.960	0.810–0.993	< 0.001
Iris							
Intra-observer	0.990	0.948–0.998	< 0.001		0.996	0.981–0.999	< 0.001
Inter-observer	0.984	0.923–0.997	< 0.001		0.996	0.980–0.999	< 0.001
Decentration measurements							
UBM							
Scleral spur							
Intra-observer	0.927	0.677–0.987	< 0.001		0.977	0.885–0.996	< 0.001
Inter-observer	0.821	0.329–0.966	0.004		0.994	0.968–0.999	< 0.001
Iris							
Intra-observer	0.921	0.651–0.986	< 0.001		0.995	0.973–0.999	< 0.001
Inter-observer	0.960	0.813–0.993	< 0.001		0.975	0.879–0.996	< 0.001

AS-OCT, anterior-segment optical coherence tomography; UBM, ultrasound biomicroscopy

Intra-observer (observer 1 versus observer 1)

Inter-observer (observer 1 versus observer 2)

*N* = 7

**Table 2:** Intra-class correlation of final Tilt and decentration measurements in comparisons between ocular anatomical references and between imaging capture technologies, considering scan positions

Iris vs. scleral spur												
Tilt measurements												
	Horizontal							Vertical				
	**Intra-class correlation**	**95% Confidence interval**		***P*** **value**				**Intra-class correlation**		**95% Confidence interval**		***P*** **value**
AS-OCT												
Intra-observer	0.968	0.844–0.994		< 0.001				0.903		0.588–0.982		< 0.001
Inter-observer	0.879	0.505–0.978		0.001				0.838		0.378–0.970		0.003
UBM												
Intra-observer	0.875	0.490–0.977		0.001				0.993		0.967–0.999		< 0.001
Inter-observer	0.552	-0.193-0.903		0.065				0.987		0.936–0.998		< 0.001
**Decentration measurements**												
UBM												
Intra-observer	0.884		0.521–0.979	0.001			0.995		0.973–0.999		< 0.001	
Inter-observer	0.943		0.737–0.990	< 0.001			0.975		0.879–0.996		< 0.001	
**UBM vs. AS-OCT**												
**Tilt measurements**												
Scleral spur												
Intra-observer	0.920	0.650–0.986		< 0.001				-0.224		-0.779-0.566		0.702
Inter-observer	0.543	-0.205-0.901		0.068				-0.020		-0.684-0.691		0.512
Iris												
Intra-observer	0.846	0.402–0.971		0.002				-0.097		-0.723-0.649		0.585
Inter-observer	0.754	0.164–0.952		0.010				-0.012		-0.680-0.695		0.503

AS-OCT, anterior-segment optical coherence tomography; UBM, ultrasound biomicroscopy

Intra-observer (observer 1 versus observer 1)

Inter-observer (observer 1 versus observer 2)

*N* = 7

### Surgical outcomes

The demographics, previous surgeries, past ocular history, and complications are shown in Table 3. The mean patient age was 67.28 **±** 6.57 years. The mean time between the last surgery and secondary IOL implantation was 7.29 ± 3.50 months. Vision, corneal topography, refraction, intraocular pressure, and pachymetry data are shown in Table 4. At 6 months postoperatively, all patients achieved a logarithm of the minimum angle of resolution (logMAR) vision of 0.24 (Snellen 20/30) or better, except for one patient who developed cystoid macular edema (CME). The mean biometric axial length was 23.42 ± 1.10 mm, prediction error was − 1.09 ± 0.47 D, and the mean SIA was 1.59 ± 0.41 D.

**Table 3:** Patient characteristics, prior surgeries, past ocular history and complications

Age/gender	Previous surgeries - number/type	Past ocular history	Complications
71/F	1, Phaco	-	-
67/F	2, Phaco, PPV	Macula on retinal detachment	-
55/M	1, Phaco, PPV	PXF, Blunt ocular trauma, glaucoma	-
70/M	1, Phaco	-	-
76/F	2, Phaco, fixation attempt	-	CME
68/F	1, Phaco	NPDR	-
64/F	2, Phaco, fixation attempt	Glaucoma	-

F, female; M, male; IOL, intraocular lens; PPV, pars plana vitrectomy; PXF, pseudoexfoliation; NPDR, non-proliferative diabetic retinopathy; CME, cystoid macular edema

**Table 4:** Longitudinal assessment of vision, corneal topography, refraction, intraocular pressure, and pachymetry

Data	Preoperative	1 Month postoperative	3 Months postoperative	6 Months postoperative	*P* value
BCVA (logMAR)	0.59 ± 0.13^*^	0.27 ± 0.16†	0.21 ± 0.11^†^	0.24 ± 0.34^†^	0.001
Corneal topography					
K1 (diopters, D)	42.54 ± 2.14	41.68 ± 1.86	42.13 ± 2.19	41.93 ± 2.02	0.166
K2 (D)	44.16 ± 2.15	43.94 ± 2.04	44.75 ± 2.36	44.92 ± 2.21	0.126
Corneal astigmatism (D)	1.62 ± 1.06	2.25 ± 1.52	2.77 ± 1.66^*^	2.99 ± 1.53^*^	0.008
Refraction					
Spherical equivalent (D)	10.84 ± 1.22^*^	-0.93 ± 0.31†	-0.82 ± 0.66†	-1.32 ± 0.44†	< 0.001
Astigmatism (D)	-1.21 ± 1.45^*^	-1.43 ± 1.02^*^	-2.64 ± 1.22†	-2.21 ± 0.82	0.020
IOP (mmHg)	16.86 ± 5.52	14.57 ± 4.58	14.57 ± 4.28	17.43 ± 6.16	0.625
Pachymetry (micra)	549.43 ± 35.41	556.29 ± 37.32	548.00 ± 29.60	547.29 ± 31.30	0.840

Data are expressed as the mean ± standard deviation

BCVA, best-corrected visual acuity; IOP, intraocular pressure; logMAR, logarithm of the minimum angle of resolution

*P* value, descriptive level of analysis of variance with repeated measures

*N* = 28 observations related to 7 patients

*†‡ indicate distinct means according to multiple comparisons with Bonferroni correction

Observer 1 measured an overall mean tilt of 1.15° ± 1.26° 6 months postoperatively in the AS-OCT images. The horizontal meridian assessment revealed a mean tilt of 1.25° ± 1.61°, with four eyes exhibiting anterior displacement of the nasal portion and three showing posterior displacement. For the vertical meridian, the mean tilt was 1.07° ± 0.94°, with four eyes displaying anterior displacement of the superior portion and three showing posterior displacement.

The final decentration measurements obtained from the UBM images showed an overall mean decentration of 0.58 ± 0.63 mm. The average horizontal decentration was 0.44 ± 0.22 mm, with six eyes showing temporal displacement and one eye showing nasal displacement of the IOL. In the vertical meridian, the average decentration was 0.73 ± 0.88 mm, with five eyes exhibiting inferior displacement and two eyes with superior displacements of the IOL. Qualitative analysis of the AS-OCT images indicated that the IOL edges were visible between lines 2 and 3 in both meridians for six of the seven patients at 1, 3, and 6 months. One patient had the horizontal meridian between lines 2 and 3, while the vertical meridian was between lines 2 and 1 (Fig. 2C).

When comparing the measurements taken during months 1 and 6 postoperatively, no significant effects over time were observed regarding the IOL positioning. In the horizontal position, the tilt (*P* = 0.592) and decentration (*P* = 0.593) did not change over time. Similarly, in the vertical position, no effects over time were seen in tilt (*P* = 0.256) and decentration (*P* = 0.063).

## Discussion

With the aims of enhancing the anatomic understanding of secondary implants and proposing a new method to measure the IOL position based on OCT and UBM images, this study evaluated the outcomes of a 4-point fixation IOL sutured to the sclera using objective measurements. Traditional assessments have relied on subjective methods, such as slit-lamp observations and built-in calipers in UBM and AS-OCT images, which often show low reproducibility [9–13].

Our findings indicated a mean overall tilt of 1.15° ± 1.26°, with a vertical tilt at 1.07° ± 0.94° and a horizontal tilt at 1.25° ± 1.61°. The current literature lacks tilt measurements specific to the 4-point scleral fixation technique for direct comparison. However, this technique shows lower tilt values compared to the 2-point techniques, such as the Yamane technique (2.3° ± 1.9°) [11], the Carlevale IOL (Soleko, Milan, Italy) (2.08° ± 1.19°) [12], the Agarwal technique (3.2° ± 2.7° horizontally and 2.9° ± 2.6° vertically) [14], and the retropupillary iris-claw technique (5.0°, range: 3.7°-6.2°) [15]. Studies have shown that physiologic tilt can increase up to a maximum of 5° before symptoms begin to manifest. Tilts exceeding this threshold can significantly affect visual quality [16].

Measuring decentration entails intricate mathematical formulas that can be challenging to implement on a large scale. Although the CASIA 2 software (Casia SS-OCT, Tomey Corporation, Nagoya, Japan) in the machine provides automatic decentration measurements, it is limited to device owners [7, 15, 17]. In this study, a straightforward calculation performed by the algorithm was used combined with qualitative assessments of the IOL decentration. The reproducibility analysis showed favorable results in both objective measurements obtained from UBM images and qualitative assessments from AS-OCT images. The mean decentration observed in UBM images was 0.58 ± 0.63 mm, predominantly in the temporal direction. Although no comparable measurements for decentration utilizing this technique exist, a study investigating retropupillary iris-claw fixation reported a median decentration of 0.67 mm (range: 0.39–0.76 mm), also exhibiting a similar infero-temporal tendency [15]. Studies have shown that the crystalline lens and IOL placed in the capsular bag show a mean physiologic decentration of 0.12 mm [17].

Furthermore, the stability assessments of the IOL through the tilt and decentration measurements during follow-up showed no significant time-related effects, implying that the IOL remains stable. This stability is crucial, particularly as other techniques are linked to serious complications such as IOL dislocation and suture erosion [10, 18, 19] Consequently, the 4-point scleral sutured technique minimizes the need for reoperation over the long term [3]. Importantly, this technique is not limited by the size of the IOL, allowing for placement of the lens further posterior to the limbus (3–4.0 mm). This positioning effectively avoids the ciliary body and helps prevent complications such as uveitis-glaucoma-hyphema syndrome.

Poorly positioned IOLs can cause dysphotopsias and diminish the quality of life, with axial tilt inducing astigmatism and decentration leading to visual aberrations such as coma [20, 21]. Thus, surgeons should be encouraged to evaluate the tilt and decentration assessing secondary IOL implants. To perform precise measurements, it is crucial to establish reliable anatomic references in the eye and IOL.

Tilt measurement requires horizontal and vertical references in the eye and the IOL. For horizontal references, the IPE plane is commonly used, particularly in UBM, along with a line connecting the SS observable in UBM and AS-OCT images [22]. Each reference has its strengths and limitations; for instance, identifying the IPE can be challenging in the presence of tissue atrophy. Additionally, the SS may be obscured in uncooperative patients with limited eyelid opening, which we experienced in one of the current cases. This restriction impacted the reproducibility of the tilt measurements from the AS-OCT vertical scans, resulting in decreased inter-examiner reliability, in that the observers estimate the SS position based on obstructed views. Consequently, this study used both references while performing an intra-class correlation analysis, which indicated comparable measurements. Thus, either reference can be adopted based on the visibility of the anatomic structures in imaging.

For the horizontal reference on the IOL, some studies have used the anterior surface of the IOL, while others refer to its horizontal axis, dividing the IOL into equal halves [7, 10–12, 18]. However, given the varying geometries of different IOLs, relying solely on the anterior surface can introduce variability and compromise measurement reproducibility. Therefore, the IOL horizontal axis was selected as a reference, enhancing reproducibility across various IOL types.

The ideal vertical reference would be the line of sight, an imaginary line linking an object in space to the pupillary center and the fovea. However, this requires an OCT system capable of scanning the entire eye, which is not presently available in clinical settings. Some studies have used the topographic apex of the cornea as a reference; however, its position varies significantly between patients and may misalign with the pupillary axis, especially in keratoconus or pellucid marginal degeneration [17]. Therefore, this reference was not used. Instead, a vertical reference was established as a line drawn perpendicularly through the midpoint of the horizontal reference, enabling determination of the center of the circle defined by the SS and/or the iris root [22].

An important finding of the current study is that the measurements obtained from UBM images were not comparable to those obtained through AS-OCT images. Thus, if a stability analysis is initiated using a specific technology, it is essential that all subsequent measurements throughout follow-up use the same technology. Conversely, the proposed algorithm allows for the analysis of any fixation type, whether situated in the anterior or posterior chamber of the eye, as long as the IOL is visible in the AS-OCT or UBM images. By using anatomical references in the images, it is possible to accurately position reference lines within the eye, at the SS or IPE, and across the optical zone of the IOL, along its horizontal axis and obtain the measurements.

The final mean logMAR BCVA achieved was 0.24 ± 0.34, similar to previous studies with the same 4-point fixation technique, which reported final mean logMAR BCVA values of 0.35 ± 0.45 [2], 0.17 ± 0.18 [4], 0.33 ± 0.36 [23], and 0.49 ± 0.52 [24]. The refractive outcomes were favorable, with most patients within ± 1 D of the target (mean − 1.32 ± 0.44 D), which agreed with findings from similar studies that reported means of -0.79 ± 0.95 D [23], -0.99 ± 1.00 D [24], and − 1.29 ± 1.76 D [3]. However, the prediction error of -1.09 ± 0.47 D was greater than in other studies and attributed to astigmatism induced intraoperatively [23, 24]. Notably, induced astigmatism of 1.59 ± 0.41 D in our series was higher than the 0.73 ± 0.59 D reported in another study, likely because our patients underwent multiple surgeries before the fixation surgery and already had poor anterior segment anatomy [20].

Surgical complications included one case of CME that was effectively treated and improved with corticosteroids and anti-inflammatory drops. The decision to perform four-point IOL fixation surgery in conjunction with vitrectomy is critical for preventing vitreous-related surgical complications. A non-associated PPV secondary implant study with large sample size, have presented a 16% rate of vitreous prolapse and a 1.5% rate of retinal detachment [25]. Vitrectomy, which involves shaving the vitreous base and removing lens or capsule remnants, prevents vitreous prolapse and entrapment, thereby reducing the risk of retinal tears, detachments, and endophthalmitis. It also facilitates better positioning of the IOL by eliminating mass effects that can tilt the lens. Even in patients with a prior vitrectomy, a second intervention is advisable to ensure these issues are effectively managed.

The strengths of our study include its prospective design, comprehensive multimodal analysis over the follow-up period, and the creation of a novel algorithm. To the best of our knowledge, this is the first study that proved objectively that the 4-point IOL fixation exhibits low tilt and decentration and remains stable over a 6-month follow-up period. Moreover, this study is pioneering in developing an innovative, simple, and machine-free method for measuring the positions of secondary implants. The ability to perform these measurements with readily available imaging represents a significant advancement, democratizing access to information, enhancing the database, and expanding our anatomic understanding of secondary implant positioning. This facilitates long-term assessments and promotes data-driven discussions regarding secondary implants.

Limitations in the study include the small sample size, short follow-up duration, and the necessity for trained physicians to perform the measurements. Despite its small size, the sample demonstrated a high statistical power (80.7% for an estimated correlation of 0.78). For intraclass correlations exceeding this value (as shown in Table 1), the power of the sample is even greater when applying the F-test. This indicates that, due to the high intraclass correlation of the measurements obtained with the algorithm, the method can be considered reliable for measuring tilt and decentration. Nevertheless, future studies should aim to include more patients and long-term measurements.

In conclusion, our study objectively showed that the scleral fixation of a 4-point IOL using Gore-Tex sutures exhibits low tilt and decentration and remains stable over a 6-month follow-up period. The study also showed favorable visual outcomes postoperatively. Furthermore, the current work establishes a foundation for standardizing positioning measurements of secondary implants. Future efforts should involve expanding the database to incorporate other currently available secondary implant techniques, enabling comprehensive comparisons of surgical methods to identify which techniques exhibit greater stability and minimal tilt or decentration over the long term.

## Electronic supplementary material

Below is the link to the electronic supplementary material.

Supplementary Material 1

## Data Availability

No datasets were generated or analysed during the current study.

## Abbreviations

IOL: Intraocular lens
logMAR BCVA: Logarithm of the minimum angle of resolution best-corrected visual acuity
AS-OCT: Anterior -segment optical coherence tomography
UBM: Ultrasound biomicroscopy
SS: Scleral spur
IPE: Iris pigmented epithelium
CME: Cystoid macular edema
D: Diopter
SIA: Surgically induced astigmatism
VRL: Vertical reference line
